# Polysomnographic and neuropsychological characteristics of rapid eye movement sleep behavior disorder patients

**DOI:** 10.1002/brb3.1220

**Published:** 2019-02-14

**Authors:** Xuan Zhang, Zhiyi Song, Jingyi Ye, Ying Fu, Jinying Wang, Lei Su, Xiaodong Zhu, Meiyun Zhang, Yan Cheng, Wei Wu, Rong Xue

**Affiliations:** ^1^ Department of Neurology Tianjin Medical University General Hospital Airport Site Tianjin China; ^2^ Department of Imaging Tianjin Academy of Traditional Chinese Medicine Affiliated Hospital Tianjin China; ^3^ Department of Neurology Tianjin Medical University General Hospital Tianjin China; ^4^ Department of Neurology Tianjin People’s Hospital Tianjin China

**Keywords:** neuropsychological performance, RBD, sleep characteristics

## Abstract

**Objectives:**

To compare the sleep characteristics and cognition between rapid eye movement sleep behavior disorder (RBD) patients and non‐RBD (nRBD) healthy control subjects and to determine the correlation between sleep and cognition in RBD patients.

**Methods:**

Polysomnography (PSG) was performed to confirm and exclude RBD. Fifteen iRBD patients, 12 PD with RBD patients, and 23 matched nRBD healthy control subjects were enrolled. Subjective sleep and neuropsychological evaluations were performed.

**Results:**

No differences were found in the subjective sleep among the three groups. Compared to the nRBD subjects, iRBD patients showed higher PLMI, the PD with RBD patients showed an increased proportion of N1 sleep, a decreased proportion of N2 and N3 sleep, and an increased periodic leg movement index. The iRBD patients performed worse on RCFT time than the nRBD subjects, the PD with RBD patients performed worse than the nRBD subjects on the following evaluations: the Mini‐Mental State Examination; auditory verbal learning test (AVLT); Rey Complex Figure Test (RCFT) time, Clock drawing test (CDT); delay memory score of RCFT; Symbol digit modalities test (SDMT); Trail Making Test (TMT); and Stroop Test B and C, all significant changes (all *p* < 0.05). Furthermore, in RBD patients, lower sleep efficiency was correlated with decreased SDMT scores (*r* = 0.694, *p* < 0.01), longer time on the TMT A (*r* = −0.589, *p* < 0.01), and lower city fluency test scores(*r* = 0.556, *p* < 0.01). Less total sleep time was correlated with lower RCFT scores (*r* = 0.392, *p* = 0.043), longer time on the TMT A (*r* = −0.417, *p* = 0.031), and lower city fluency test scores (*r* = 0.405, *p* = 0.036).

**Conclusions:**

PD with RBD patients suffered from abnormal sleep architecture and extensive cognition dysfunction. Decreased total sleep time and sleep efficiency may contribute to cognitive deterioration in RBD patients.

## 1 INTRODUCTION

Rapid eye movements, cortical activation, vivid dreaming, skeletal muscle paralysis (atonia), and muscle twitches are the characteristics of rapid eye movement (REM) sleep. Loss of normal muscle atonia during REM sleep leads to a parasomnia called REM sleep behavior disorder (RBD). During REM sleep, RBD patients act out their dreams violently and forcefully, which may lead to disturbed sleep or injuries to themselves or their bed partners.

RBD may be either idiopathic or secondary to other functional or structural disorders of the nervous system. The prevalence of RBD is 0.38%–2.01% in the general population but is much higher in patients with neurodegenerative diseases, especially synucleinopathies (Jiang et al., 2017). The estimated risk of an identified neurodegenerative syndrome from the diagnosis of idiopathic RBD (IRBD) was found to be 15%, 25%, 36%, and 41% at 2, 3, 4, and 5 years, respectively. Additionally, data from another study indicated estimated risk rates of 34.8%, 73.4%, and 92.5% at 5, 10, and 14 years, respectively. IRBD patients have a high risk of developing α–synucleinopathies, such as Parkinson's disease (PD), dementia with Lewy bodies (DLB), or multiple system atrophy (MSA).(Hogl, Stefani, & Videnovic, 2018; Iranzo et al., 2014, 2013).

There are many controversial results regarding the sleep characteristics of RBD patients as recorded by polysomnography (PSG) (Fantini et al., 2011; Ferini‐Strambi et al., 2004; Zhou et al., 2014). The principal cognitive deficits of RBD patients involve visuospatial skills, attention, execution, and memory (Hogl et al., 2018). Moreover, studies have suggested that mild cognitive impairment (MCI) in RBD may be an early marker of neurodegeneration (Gagnon et al., 2009; Sasai, Matsuura, & Inoue, 2013). However, few studies have examined the features and quality of sleep in RBD patients. It is well‐known that sleep plays an important role in memory consolidation. Sleep duration, timing, and continuity can affect cellular ultrastructure, gene expression, metabolic and hormone regulation, mood, and vigilance (Van Someren et al., 2015).

Few studies have focused on the correlation between sleep characteristics and cognition in RBD patients. However, the question of whether disrupted sleep contributes to cognitive impairment in RBD patients remains unanswered. Therefore, the aim of this study was to compare the sleep characteristics and neuropsychological performances of Chinese RBD patients using overnight video PSG and sleep and neuropsychological evaluations focusing on the following: (a) the characteristics of sleep and cognition in iRBD and PD with RBD patients; (b) the correlation between sleep and cognition in RBD patients.

## 2 PATIENTS AND METHODS

### 2.1 Subjects

Participants were recruited from Tianjin Medical University General Hospital from October 2014 to May 2017. The RBD patients were diagnosed according to the standards described in the International classification of sleep disorders (ICSD‐3) criteria (Sateia, 2014). Patients with the following conditions were excluded: (a) hearing loss, parachromatism, illiteracy, and the inability to complete the required tests; (b) a history of psychiatric diseases such as depression and schizophrenia; (c) severe medical conditions; (d) cerebrovascular disease, neurological conditions, a history of head trauma, or sleep disorders such as parasomnia or obstructive sleep apnea syndrome; and (e) treatment with a drug which known to influence REM sleep. Fifteen iRBD and 12 PD with RBD consecutive patients and 23 age‐, gender‐ and education‐matched healthy controls were enrolled. We obtained signed agreement forms from all of the participants. The study was approved by the Tianjin Medical University General Hospital Review Board and Ethics Committee. The main demographic and clinical characteristics, including age, gender, and disease duration, were recorded for each patient.

### 2.2 Polysomnography examination

All the subjects were observed with overnight video PSG using a digital sleep laboratory system (Nicolet v32, Natus Medical Incorporated, Pleasanton, CA). Participants were instructed to go to sleep at their usual bedtimes. All PSG sessions were monitored by a trained technician according to standardized criteria (Hakkinen et al., 1993). The following sleep variables were acquired and analyzed: sleep latency (SL); REM SL; sleep efficiency (SE); total sleep time (TST); the percentage of stage N1, N2, N3, and REM sleep; the apnea–hypopnea index (AHI); the periodic leg movement index (PLMI); average SpO_2_; and minimum SpO_2_.

### 2.3 Subjective sleep and neuropsychological evaluations

Subjective sleep and neuropsychological evaluations were performed by a trained doctor in an evaluator‐blinded fashion. The sleep evaluation included the following: (a) daily sleepiness was evaluated with the Epworth Sleepiness Scale and (b) nocturnal sleep disorders and diurnal drowsiness were evaluated with the Scales for Outcomes in PD (SCOPA)‐Sleep scale. The neuropsychological evaluation included the following: (a) general cognitive functioning was evaluated with the Chinese version of the Mini‐Mental State Examination (MMSE) (Cui et al., 2011); (b) verbal memory was evaluated with the Auditory Verbal Learning test, including the total score and the short delay recall, long delay recall, and recognition error scores (Guo, Zhao, Chen, Ding, & Hong, 2009); (c) visuospatial skills were evaluated with the Rey Complex Figure Test (RCFT) (copy and time) (Caffarra, Vezzadini, Dieci, Zonato, & Venneri, 2002) and the Clock drawing test (Rouleau, Salmon, Butters, Kennedy, & McGuire, 1992), and visual memory was evaluated with the RCFT delay memory score(Caffarra et al., 2002); (d) verbal information processing was evaluated with the Symbol Digit Modalities Test (SDMT) (Strauss, Sherman, & Spreen, 2006); (e) executive function was evaluated with the Trail Making Test (TMT) A (Straus et al., 2006), TMT B (Cheung, Cheung, & Chan, 2004), Stroop Color‐Word Test (Strauss et al., 2006) (modified version) (Stroop A, Stroop B, and Stroop C), and Stroop interference effect (equal to the time of Stroop C minus the time of Stroop B); and (f) language was evaluated according to semantic verbal fluency (Strauss et al., 2006) (animal fluency test, city fluency test, and animal‐city alternation fluency test) and naming ability [the Boston Naming Test (Chiu et al., 2000)].

### 2.4 Statistical analysis

The results are expressed as the mean ± standard error (SE) for continuous variables and as the probability (percent) for categorical variables. Differences in the demographic and clinical characteristics among the three groups were compared descriptively using chi‐square tests for categorical measures; using one‐way ANOVA tests for data that were normally distributed and which homoscedasticity was respected; using nonparametric Kruskal–Wallis or Mann–Whitney *U* tests for variables that were not distributed normally or for which homoscedasticity was not respected. Neuropsychological test scores and PSG parameters in RBD participants were converted into *z* scores using the mean and SDs of the RBD participants’ scores before correlation analysis. Linear models were applied to compute the Pearson correlation coefficient (*r*) between sleep and neuropsychological parameters in the RBD group. Statistical significance was defined as *p* < 0.05. The statistical analyses were performed using SPSS 16.0. Figures were created using GraphPad Prism Version 5 (San Diego, USA).

## 3 RESULTS

### 3.1 Demographics, self‐report questionnaires, and overnight sleep studies of the three groups

The demographic and clinical characteristics are reported in Table 1. There were no differences in age, gender, or education between the three groups. The average RBD duration of iRBD and PD with RBD patients was 5.77 and 11.46 years, respectively.

**Table 1:** Demographic and clinical characteristics of RBD and nRBD

	iRBD (*n* = 15)	PD + RBD (*n* = 12)	nRBD (*n* = 23)	*p*
Age, year	64.93 ± 1.81	68.83 ± 2.98	63.39 ± 2.14	NS
Female, number (%)	8 (53.3)	7 (58.3)	13 (56.5)	NS
Education, year	9.13 ± 0.59	9.08 ± 1.12	10.96 ± 0.62	NS
RBD duration, year	5.77 ± 1.40	11.46 ± 3.99	–	NS

Note

Plus‐minus values are means ± SEM.

The results from the self‐report questionnaires and polysomnographic parameters are reported in Table 2. The subjective sleep evaluations indicated no significant differences in daytime sleepiness, night insomnia, and quality of sleep among the three groups. PD with RBD patients had a higher proportion of N1 (32.33 vs. 15.61, *p* < 0.01), lower proportion of N2 (52.0 vs. 63.39, *p* < 0.01), lower proportion of N3 (2.92 vs. 6.87, *p* < 0.05) than the nRBD healthy control subjects. IRBD patients had a higher PLMI than PD with RBD patients and nRBD healthy control subjects (26.55 vs. 13.58 vs. 4.87, *p* < 0.05, *p* < 0.01), lower proportion of N1 than PD with RBD patients (19.93 vs. 32.33, *p* < 0.05). No differences were found in the TST, SL, REM SL, SE, proportion of REM sleep, arousal index, average SpO_2_, minimum SpO_2_, or AHI among the three groups.

**Table 2:** Objective and subjective sleep characteristics of RBD and nRBD

	iRBD (*n* = 15) (A)	PD + RBD (*n* = 12) (B)	nRBD (*n* = 23) (C)	*p*
Objective evaluation (v‐PSG)				
TST, min	376.10 ± 15.05	365.75 ± 24.89	392.54 ± 12.38	NS
SL, min	10.033 ± 1.31	10.71 ± 2.00	19.63 ± 5.53	NS
REML, min	149.3 ± 29.54	156.79 ± 23.20	159.83 ± 18.89	NS
SE, %	71.15 ± 3.47	73.26 ± 4.64	75.83 ± 2.52	NS
N1, %	19.93 ± 2.43	32.33 ± 3.86	15.61 ± 1.929	A ＜ B* C ＜ B**
N2, %	60.33 ± 2.58	52.0 ± 3.87	63.39 ± 1.81	B ＜ C**
N3, %	5.67 ± 1.39	2.92 ± 1.46	6.87 ± 1.19	B ＜ C**
REM, %	14.2 ± 1.549	12.75 ± 2.10	13.09 ± 1.14	NS
Arousal index, /hr	10.24 ± 2.28	8.37 ± 1.45	9.82 ± 1.64	NS
PLM index, /hr	26.55 ± 5.99	13.58 ± 4.91	4.87 ± 1.70	A ＞ B* A ＞ C**
Average SpO_2_, %	94.80 ± 0.44	94.83 ± 0.46	94.00 ± 0.37	NS
Minimum SpO_2_, %	89.33 ± 0.91	91.17 ± 0.53	83.83 ± 3.72	NS
AHI	3.88 ± 1.262	3.25 ± 1.46	6.47 ± 1.88	NS
Subjective evaluation				
ESS	5.80 ± 1.63	6.58 ± 1.32	4.70 ± 0.48	NS
Scopa‐sleep				
Daily sleepiness	3.27 ± 0.43	3.0 ± 0.536	2.869 ± 0.34	NS
Night insomnia	2.87 ± 0.86	5.00 ± 1.26	4.91 ± 0.92	NS
Quality of sleep	3.27 ± 1.25	2.25 ± 0.55	1.35 ± 0.30	NS

Note

Plus‐minus values are means ± SEM.

TST: total sleep time; SL: latency to sleep onset; REML: latency to rapid eye movement; SE: sleep efficiency; REM: rapid eye movement; PLM: periodic leg movement; SpO_2_: serum oxygen saturation; AHI: apnea‐hypopnea index; ESS: Epworth Sleepiness Scale; NS: not significant.

* *p* ＜ 0.05

** *p* ＜ 0.01.

### 3.2 Cognitive deficits in the iRBD group and PD with RBD group

The iRBD patients showed deficit on RCFT time (*p* = 0.043). PD with RBD patients had significantly lower scores than the nRBD healthy control subjects on the following evaluations: the MMSE (*p* = 0.008); AVLT total(*p* = 0.021), AVLT short delay recall (*p* = 0.001), AVLT long delay recall of (*p* = 0.026); RCFT time (*p* = 0.043), CDT (*p* = 0.033); delay memory score of RCFT (*p* = 0.027); SDMT (*p* = 0.037); TMT A (*p* = 0.002); TMT B (*p* = 0.006); Stroop Test B (*p* = 0.006); Stroop Test C (*p* = 0.003); Stroop interference effect (*p* = 0.046). The domains that demonstrated cognitive deficits in PD with RBD patients included general cognition, verbal memory, visuospatial abilities, and memory, verbal information processing and execution. PD patients with RBD performed worse than iRBD patients on AVLT short delay recall (*p* = 0.042) and TMT A (*p* = 0.037). The details of the neuropsychological tests are summarized in Table 3.

**Table 3:** Neuropsychological analyses of RBD and nRBD

	iRBD (*n* = 15) (A)		PD+RBD (*n* = 12) (B)	nRBD (*n* = 23) (C)	*p*
MMSE	27.40 ± 0.73		25.58 ± 1.04	28.09 ± 0.31	B ＜ C**
Verbal memory					
AVLT					
Total	15.33 ± 1.27		13.67 ± 1.13	17.74 ± 1.06	B ＜ C*
Short‐delay memory	5.47 ± 0.58		3.67 ± 0.40	6.26 ± 0.47	B ＜ C** B ＜ A*
Long‐delay memory	4.47 ± 0.62		4.08 ± 0.49	5.87 ± 0.48	B ＜ C*
Recognition	20.33 ± 0.70		19.25 ± 0.46	20.91 ± 0.63	NS
Visuospatial abilities					
Rey Complex Figure Test					
Score	27.20 ± 2.25	26.50 ± 2.85		31.57 ± 1.18	NS
Time	272.00 ± 32.77	286.58 ± 35.34		198.63 ± 16.54	B ＞ C* A ＞ C*
CDT	2.73 ± 0.15	2.33 ± 0.33		2.87 ± 0.07	B ＜ C*
Visuospatial memory					
Rey Complex Figure Test delay memory score	11.67 ± 1.38		7.33 ± 1.74	12.78 ± 1.61	B ＜ C*
Verbal information processing					
Symbol digit modalities test	25.87 ± 3.00		19.92 ± 3.68	29.70 ± 2.83	B ＜ C*
Executive function					
TMT					
TMT A	93.53 ± 8.96		129.17 ± 19.94	78.80 ± 5.84	B ＞ C** B ＞ A*
TMT B	247.87 ± 29.42		279.08 ± 26.52	192.50 ± 10.73	B ＞ C**
Stroop test					
Stroop A	21.13 ± 1.88		22.75 ± 2.91	18.19 ± 0.86	NS
Stroop B	27.73 ± 1.88		32.50 ± 4.31	22.97 ± 1.34	B ＞ C**
Stroop C	42.00 ± 2.21		47.42 ± 4.52	35.05 ± 2.07	B ＞ C**
Stroop interference effect	18.13 ± 2.39		14.92 ± 3.00	12.08 ± 1.63	B ＞ C*
Language					
Verbal fluency test					
Animal	16.20 ± 1.23		15.50 ± 1.86	17.96 ± 0.77	NS
City	16.47 ± 1.26		13.58 ± 1.04	16.65 ± 0.93	B ＜ C*
Animal‐city alternation	10.13 ± 1.12		7.00 ± 0.64	8.52 ± 0.60	NS
Boston naming test	19.20 ± 0.48		18.33 ± 0.48	19.22 ± 0.22	NS

Note

Plus‐minus values are means ± SEM.

MMSE: Mini‐Mental State Examination; AVLT: auditory verbal learning test; CDT: clock drawing test; TMT: trial making test; NS: not significant.

* *p* ＜ 0.05;

** *p* ＜ 0.01.

### 3.3 Correlation between sleep parameters and neuropsychological parameters in the RBD group

**Figure 1:**
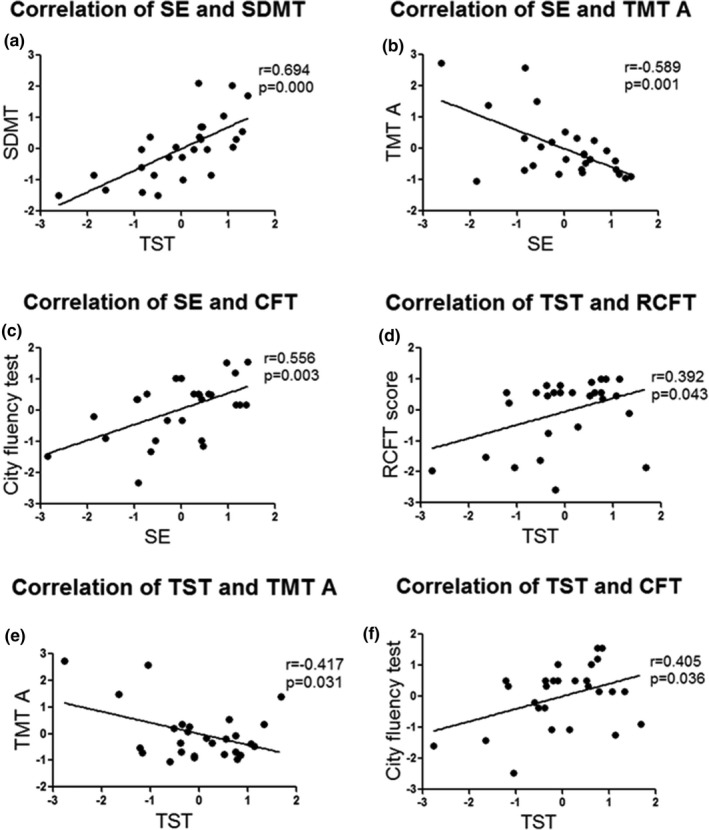
Correlation between sleep characteristic and neuropsychological parameters in **RBD group: (a) Lower SE was correlated with decreased SDMT scores (*r* = 0.694, *p* < 0.01); (b) Lower SE was correlated with longer time on the TMT A (*r* = 0.589, *p* < 0.01); (c) Less SE was correlated with lower city fluency test scores (*r* = 0.556, *p* < 0.01); (d) Lower TST was correlated with lower RCFT scores (*r* = 0.392, *p* = 0.043); (e) Lower TST was correlated with longer time on the TMT A (*r* = −0.417, *p* = 0.031); (f) Lower TST was correlated with lower city fluency test scores (*r* = 0.405, *p* = 0.036). SE: sleep efficiency; SDMT: Symbol digit modalities test; TMT: trial making test; TST: total sleep time; CFT: city fluency test; RCFT: Rey Complex Figure Test

Lower sleep efficiency was correlated with decreased SDMT scores (*r* = 0.694, *p* < 0.01), longer time on the TMT A (*r* = 0.589, *p* < 0.01), and lower city fluency test scores(*r* = 0.556, *p* < 0.01). Less total sleep time was correlated with lower RCFT scores (*r* = 0.392, *p* = 0.043), longer times on the TMT A (*r* = −0.417, *p* = 0.031), and lower city fluency test scores (*r* = 0.405, *p* = 0.036) (Figure 1).

## 4 DISCUSSION

Our study demonstrated the following results: (a) sleep architecture was disrupted in patients with PD with RBD patients, as indicated by the increased percentage of N1, the decreased percentage of N2 and N3, and the higher PLMI; (b) iRBD showed higher PLMI than the nRBD healthy control subjects; (c) PSG examination could be used to detect abnormal sleep architecture in RBD patients before subjective complaints; (d) PD with RBD patients performed worse than the nRBD healthy control subjects in terms of general cognition, verbal memory, visuospatial abilities and memory, verbal information processing, execution and language; iRBD patients showed dysfunction in visuospatial abilities; PD patients with RBD performed worse than iRBD patients in verbal memory and executive function; (e) lower SE was correlated with worse performance in verbal information processing, execution and language fluency, and lower TST was correlated with poor visuospatial abilities, execution and language fluency in RBD patients; and (f) though cognitive impairment in RBD patients may be an early symptom of neurodegenerative diseases, the possible contribution of sleep disturbance to cognitive deterioration in RBD patients should not be neglected. Sleep disruption in RBD patients should be treated in clinic and managed if possible.

In this study, RBD patients were recruited consecutively in the Department of Neurology of a university‐affiliated hospital over the span 2 years. The controls were recruited from the health examination center of the same hospital. During this time, we conducted the PSG, neuropsychological and subjective sleep evaluations of the patients and controls systematically in a clinical setting. We believe that our data are representative of the polysomnographic and neuropsychological characteristics of RBD patients.

Polysomnographic characteristics in RBD patients are controversial. Our data are consistent with those of a previous study of Chinese RBD patients that showed similar results, with more stage N1 sleep, less stage N2 and N3 sleep, and a higher PLMI than observed in the controls (Zhou et al., 2014). The SE of the RBD group appeared to be less than that of the nRBD group, but there was no significant difference between the two, most likely due to the small sample size. In contrast, a reanalysis found that the percentage of slow‐wave sleep (SWS) increased in RBD patients (Schenck, Callies, & Mahowald, 2003). The younger iRBD group (younger than 70 years) showed a significantly lower proportion of N3, while the older iRBD group (70 years or older) showed a significantly higher proportion of N3 than the control patients (Sasai et al., 2013). Sleep changes with age, as indicated by reductions in electroencephalographic slow‐wave activity and spindle frequency activity and increases in involuntary awakening (Cajochen, Munch, Knoblauch, Blatter, & Wirz‐Justice, 2006). Thus, the age of a subject should be considered in regard to the differing results. In addition, a recent study showed that 21 RBD patients demonstrated more stage N2 sleep, less REM sleep, and a lower AHI during REM sleep than 1,629 RBD negative subjects (Haba‐Rubio et al., 2017). There have also been some studies with small sample sizes that did not find differences between iRBD patients and controls in polysomnographic characteristics, except in the PLMI (Fantini et al., 2011; Ferini‐Strambi et al., 2004) and sleep duration (Massicotte‐Marquez et al., 2008). IRBD is considered a possible marker of neurodegenerative diseases and does not include RBD combined with neurodegenerative diseases. In our study, no difference was found in sleep architecture between iRBD and nRBD subjects, while PD with RBD patients showed more disturbed sleep architecture than the iRBD patients. Serious sleep disturbance in PD with RBD patients may be associated with the extensive neurodegenerative changes in the sleep regulating structures. In addition, the high PLMI in RBD patients may lead to more sleep fragmentation and decreases in sleep quality. Sleep‐wake rhythm disturbances, respiratory dysrhythmias, and parasomnias, particularly RBD, occur frequently in neurodegenerative diseases (Raggi & Ferri, 2010). Previous studies of the relationship between circadian/sleep disruption and neurodegenerative disorders have supported that neurodegeneration may impact the brain centers that control sleep and circadian behavior; sleep and circadian disruption may also lead to oxidative damage, metabolic disruption, and decreased clearance of metabolites, such as β‐amyloid, all of which may accelerate neurodegeneration (Mattis & Sehgal, 2016). Thus, sleep disruption in RBD patients should be treated in the clinic and managed if possible.

Cognitive deficits in RBD have been frequently reported in previous studies. MCI was found in 50% of iRBD patients and 73% of PD patients with RBD, whereas this condition was observed in only 11% of PD patients without RBD and 8% of control patients (Gagnon et al., 2009). RBD is an important risk factor for MCI, as deficits in executive function, verbal delayed memory and visuospatial function have been consistently associated with more severe RBD symptoms (Zhang et al., 2016). IRBD patients have been shown to perform more poorly on the word span, Rey‐Osterrieth Complex Figure Recall, digit span and logic memory tests, and on visuo‐constructional learning, sharing common features of cognitive deficits with Lewy body disease in particular (Terzaghi et al., 2008). But we found that only iRBD patients performed worse on RCFT time than the nRBD healthy control subjects, the reason may be that the RBD duration of iRBD patients is shorter in our study. The deterioration in nonverbal logic, attention, executive function and memory observed in RBD follow‐ups suggests an underlying evolution of the degenerative process (Terzaghi, Zucchella, Rustioni, Sinforiani, & Manni, 2013; Youn et al., 2016). In our study, multidomain cognitive impairment, including in verbal memory, visuospatial abilities and memory, verbal information processing, execution and language, was found in PD with RBD patients, more extensive domains of impaired cognition than iRBD patients. This indicated that PD with RBD patients exhibit more serious and extensive neurodegeneration from brainstem to mesocortex and neocortex than iRBD patients. Several pathological bases of cognitive impairment in RBD patients have been proposed. First, cholinergic dysfunction is thought to be associated with cognitive decline in iRBD (Nardone et al., 2012). Second, many imaging studies have supported abnormalities in both structure and function in brain regions other than the brainstem. Decreased GMV of the left posterior cingulate, hippocampus (Lim et al., 2016) and superior frontal sulcus (Rahayel et al., 2015) and decreased cortical thickness in the frontal cortex, lingual gyrus, and fusiform gyrus have been detected in RBD patients. Cortical hypoperfusion in the occipital, temporal, and parietal regions in RBD patients and MCI was detected using single‐photon emission computed tomography (Vendette et al., 2012). Furthermore, cortical and subcortical GM abnormalities are associated with cognitive status in patients with RBD, with more extensive patterns in patients with MCI (Rahayel et al., 2018). Imaging abnormalities in RBD patients explain the impairment in information extraction, visuospatial abilities and executive function. Third, lack of delta‐band functional connectivity (Sunwoo et al., 2017) and electroencephalographic slowing, especially during REM sleep (Sasai et al., 2013), is associated with cognitive decline in iRBD.

However, no studies have considered that disturbed sleep may lead to deterioration in cognition among RBD patients. The relationship between sleep disorders and cognition has been widely studied. Sleep promotes learning‐dependent synapse formation and maintenance of selected dendritic branches, both of which contribute to memory storage (Yang et al., 2014). Convective fluxes in interstitial fluid have been shown to increase the rate of β‐amyloid clearance during sleep (Xie et al., 2013). Moreover, the production of soluble Aβ may be relatively accelerated during sleep due to the reduced SWS in the context of aging and associated sleep disturbances. Disturbed sleep results in increased time awake during the relative sleep period and may link Aβ pathology with hippocampal‐dependent cognitive decline (Lucey & Bateman, 2014). Chronic restriction or fragmentation significantly inhibits cell proliferation and, in some cases, neurogenesis (Mueller, Meerlo, McGinty, & Mistlberger, 2015). A meta‐analysis showed that poor sleep in PD is associated with effects on memory and executive function (Pushpanathan, Loftus, Thomas, Gasson, & Bucks, 2016). However, there has been no study regarding the association between sleep abnormality and cognitive impairment. In our study, PD with RBD patients experienced less TST and SE (though not significant), decreased N2 sleep and SWS, and increased N1 sleep and PLMI, indicating that sleep quality in PD with RBD patients is worse than that in nRBD subjects. Sleep spindles are the characteristic feature of N2. A higher PLMI may induce microarousal and sleep fragmentation. Sleep spindles, SWS and REM sleep all play important roles in memory consolidation during sleep (Rasch & Born, 2013). A correlational analysis showed that lower SE was correlated with worse performance in verbal information processing, execution and language fluency, and TST was correlated with visuospatial abilities, execution and language fluency in RBD patients. Based on previous studies of sleep and cognition, we speculated that sleep abnormalities in RBD patients may contribute to cognitive deterioration.

There were some limitations to our study. First, the sample size of the study was small; thus, differences in some of the PSG and neuropsychological parameters may not have been detected. Second, this was a cross‐sectional study. A study with a large sample and a longitudinal study should be conducted to obtain more reliable and robust findings in the future.

## 5 CONCLUSION

Our study identified increased N1 sleep, decreased N2 and N3 sleep, a higher PLMI and impairments in multidomain cognitive functions, including general cognition, verbal memory, visuospatial abilities and memory, verbal information processing, execution and language, in PD with RBD patients. The sleep disturbance was more serious and the cognitive impairment was more widespread than the iRBD patients. In RBD patients, lower SE was correlated with worse performance in verbal information processing, execution and language fluency, and TST was correlated with visuospatial abilities, execution and language fluency. RBD, as a type of sleep disorder, also combines with sleep architecture disturbances and decreased sleep quality. PSG examinations can be used to detect early sleep abnormalities in RBD. Finally, while cognitive impairment in RBD patients may be an early marker of neurodegenerative diseases, the possible contribution of sleep disturbance to cognitive deterioration in RBD patients should not be neglected. Thus, sleep disruption in RBD patients should be treated in the clinic and managed if possible.

## CONFLICTS OF INTEREST

None.
